# The effect of large channel-based foraminoplasty on lumbar biomechanics in percutaneous endoscopic discectomy: a finite element analysis

**DOI:** 10.1186/s13018-024-04870-1

**Published:** 2024-07-12

**Authors:** Wei Sun, Duohua Li, Sicong Zhao, Hao Fu, Jiayu Tian, Feng Zhang, Hu Feng, Dongying Wu

**Affiliations:** 1 Department of Spine Surgery, Affiliated Hospital of Xuzhou Medical University, No. 99 Huaihai West Road, Xuzhou, Jiangsu 221006 China; 2 Graduate School of Xuzhou, Medical University, No. 209 Tongshan Road, Xuzhou, Jiangsu 221004 China

**Keywords:** Finite element analysis, Biomechanical, Range of motion, Foraminoplasty

## Abstract

**Background:**

This study aimed to evaluate the effect of foraminoplasty using large-channel endoscopy during TESSYS on the biomechanics of the lumbar spine.

**Methods:**

A complete lumbar spine model, M1, was built using 3D finite elements, and models M2 and M3 were constructed to simulate the intraoperative removal of the superior articular process of L5 using a trephine saw with diameters of 5 mm and 8.5 mm, respectively, and applying normal physiological loads on the different models to simulate six working conditions—anterior flexion, posterior extension, left-right lateral bending, and left-right rotation—to investigate the displacement and facet joint stress change of the surgical segment, and the disc stress change of the surgical and adjacent segments.

**Results:**

Compared with the M1 model, the M2 and M3 models showed decreased stress at the L4-5 left FJ and a significant increase in stress at the right FJ in forward flexion. In the M2 and M3 models, the L4-5 FJ stresses were significantly greater in left lateral bending or left rotation than in right lateral bending or right rotation. The right FJ stress in M3 was greater during left rotation than that in M2, and that in M2 was greater than that in M1. The L4-5disc stress in the M3 model was greater during posterior extension than that in the M1 and M2 models. The L4-5disc stress in the M3 model was greater in the right rotation than in the M2 model, and that in the M2 model was greater than that in the M1 model.

**Conclusion:**

Foraminoplasty using large-channel endoscopy could increase the stress on the FJ and disc of the surgical segment, which suggested unnecessary and excessive resection should be avoided in PTED to minimize biomechanical disruption.

## Introduction

Lumbar disc herniation (LDH) is a common and frequent disease in spinal surgery that seriously affects patients’ quality of life and imposes a heavy economic burden on their families and society [1, 2]. Patients with LDH who fail to respond to strict conservative treatment require surgical treatment. Traditional open surgery requires extensive incisions of the lumbar muscles and vertebral plate, and postoperative epidural scar formation is prone to cause adhesions between the dura and nerve roots, resulting in renewed compression of the dural sac, restricted nerve root movement, and possible recurrence of clinical symptoms or even surgical failure [3, 4]. A study by Ross et al. [5] revealed a significant correlation between postoperative radicular symptoms and epidural scar formation, with as many as 24% of patients suffering from scar formation resulting in prolonged postoperative pain in the lumbar spine.

The emergence of percutaneous endoscopic transforaminal discectomy (PETD) is epoch-making, and the technique has become widely popular in the last decade [6–8]. Depending on the size of the patient’s intervertebral foramen and the location of the herniated disc, intraoperative foraminoplasty is often required in PETD [9–11]. During the process of foraminoplasty, the superior articular process and synchondral ligaments are damaged to varying degrees. The lumbar facet joint (FJ) is a synovial joint that consists of a synovial membrane, joint capsule, joint fluid, and articular cartilage structures [12]. FJs play a role in restricting and guiding the movement of the spine and effectively prevent discs from being damaged by excessive shear and torsional tension. Therefore, angular changes, intraoperative injuries or even resection of the lumbar articular process may accelerate the surgical segment degeneration or even lead to degenerative spinal disease [13].

In recent years, large-channel endoscopes have become increasingly widely used in clinical practice [14, 15]. Large-channel endoscopes have a wider working channel than ordinary endoscopes and allow use of larger surgical instruments, which are minimally invasive and highly efficient. Large-channel endoscopes are usually combined with the use of an 8.5-mm-diameter trephine, which can enlarge the working space of endoscopy, making it easier to expose protruded disc, dural sac and nerve roots, but causing more damage to the FJ compared with ordinary endoscopes. To our knowledge, however, there are few studies investigating the effect of large-channel endoscopes-based foraminoplasty on lumbar biomechanics in percutaneous endoscopic discectomy.

In the present study, the authors employed the three-dimensional finite element method to simulate ordinary endoscopes-based and large-channel endoscopes-based foraminoplasty respectively on L5 superior articular process in lumbar percutaneous endoscopic discectomy, hereby to study the effect of large-channel endoscopes-based foraminoplasty on the biomechanics of operating segment and adjacent segments in percutaneous endoscopic lumbar discectomy.

## Materials and methods

### Experimental materials

#### Experimental subjects

One healthy male adult volunteer (35 years old, height 178 cm, weight 75 kg, no history of previous spinal diseases or spinal trauma) was selected for physical examination. Lumbar spine positive and lateral, hyperextension and hyperflexion films were used to exclude spinal pelvic deformity and other spinal diseases. Informed consent was signed, discussed and approved by the Ethics Committee of the Affiliated Hospital of Xuzhou Medical University.

#### Imaging equipment

A Siemens 64-slice spiral CT scanner (Siemens Sensation Open CT scanner, Siemens, Erlangen, Germany) provided by the Department of Imaging of the Affiliated Hospital of Xuzhou Medical University was used.

### Establishment of the normal L1-S1 3D finite element model

#### CT image processing and 3D image reconstruction of the L1-S1 vertebrae

The patient’s lumbar spine CT data was imported into Mimics in DICOM format, the segmentation threshold was set to 226–1612, the region growth mode was set to “6-connectivity”, and the model was optimized by mask separation, fill and mask separation. When generating the 3D geometric model, select the “high” quality level. After the 3D model is obtained, the smoothing process is carried out, the “Iterations” is set to 5, and the “Smooth factor” is set to 0.4.

#### Mesh the 3D model of theL1-S1 vertebrae

**Fig. 1:**
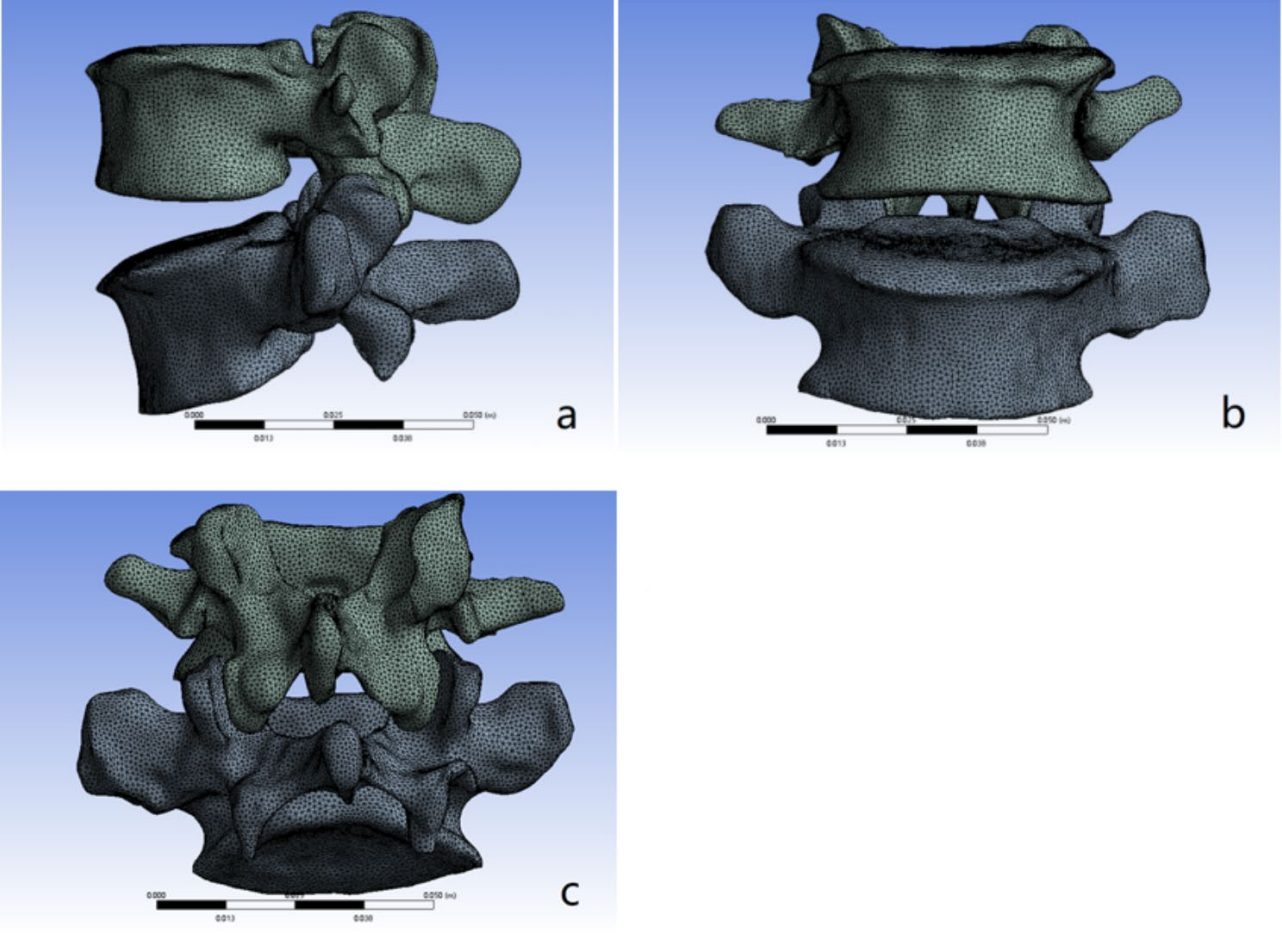
Meshing of the 3D finite element model. **a**: side view; **b**: front view; **c**: back view

Using the remesh tool to enter the Mimics comes with the Magics plug-in, showing the vertebra 3D solid surface mesh division, Magics has a powerful face mesh redrawing (remesh) function, the use of which the Smoothing tool to smooth the face mesh processing, and then use the Triangle reduction to remove some of the quality of the low Then use Triangle reduction to remove some of the poor quality triangular surface pieces, and then observe the surface of the model and manually edit and eliminate the surfaces that are still of poor quality. The modified face mesh model is shown in Fig. 1.

#### Modeling process of each intervertebral disc, articular cartilage, ligament and joint capsule in the model

**Fig. 2:**
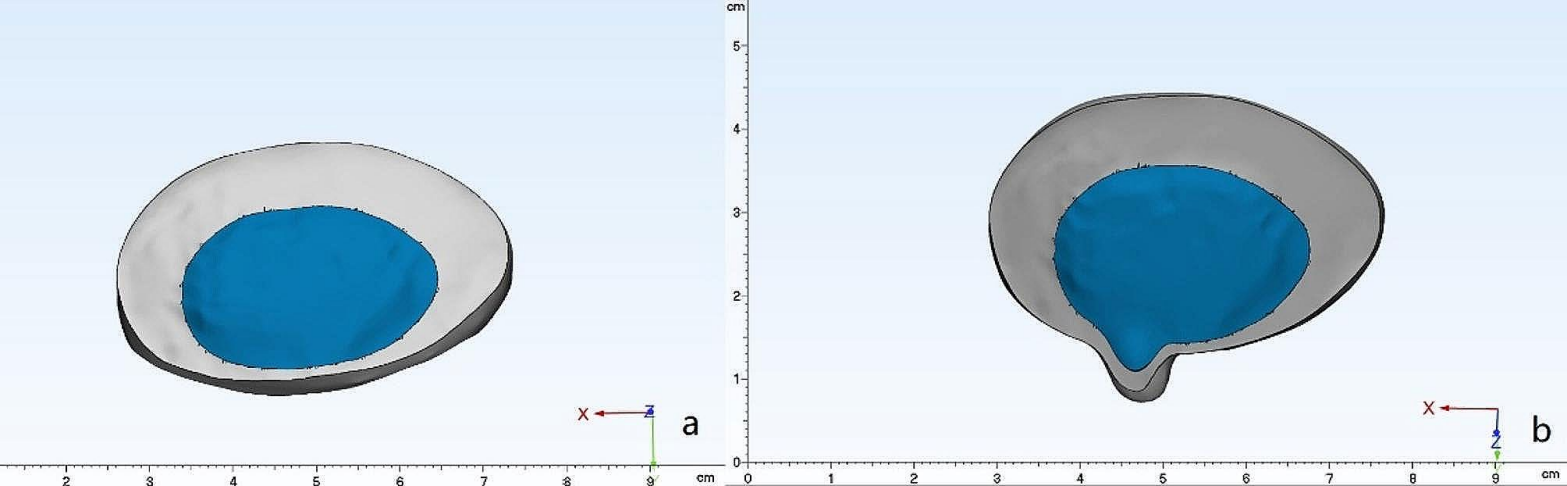
Simulation of a herniated disc. **a**: normal disc; **b**: herniated disc

The anterior longitudinal ligament, posterior longitudinal ligament, supraspinous ligament, interspinous ligament, articular capsule ligament, and intertransverse ligament were constructed in the model using three-dimensional rod units. According to the literature [16], endplates were created on the upper and lower surfaces of the vertebral body with a thickness of 1 mm. between the upper and lower endplates was the intervertebral disc, which consisted of the annulus fibrosus and the nucleus pulposus (Nucleus pulposus). The nucleus pulposus was first added between the upper and lower endplates, and then four 1.5-mm-thick concentric rings were added around the nucleus pulposus to form the annulus fibrosus. The fibers of the annulus fibrosus are constructed from rod units that can only withstand tension, and in the annulus the fibers travel in a scissor-like pattern and form an average angle of 25° to 40° to the disc. The nucleus pulposus accounts for approximately 44% of the disc volume, and the center of the nucleus pulposus is located approximately 3.5 mm behind the center of the disc. The boundary of the intervertebral discs was offset inward by 12 mm, and then its center is displaced backward by 3.5 mm, and the resulting is the boundary of the nucleus pulposus (Fig. 2). The articular cartilage is created between the upper and lower articular processes.

#### Optimization of the model

**Fig. 3:**
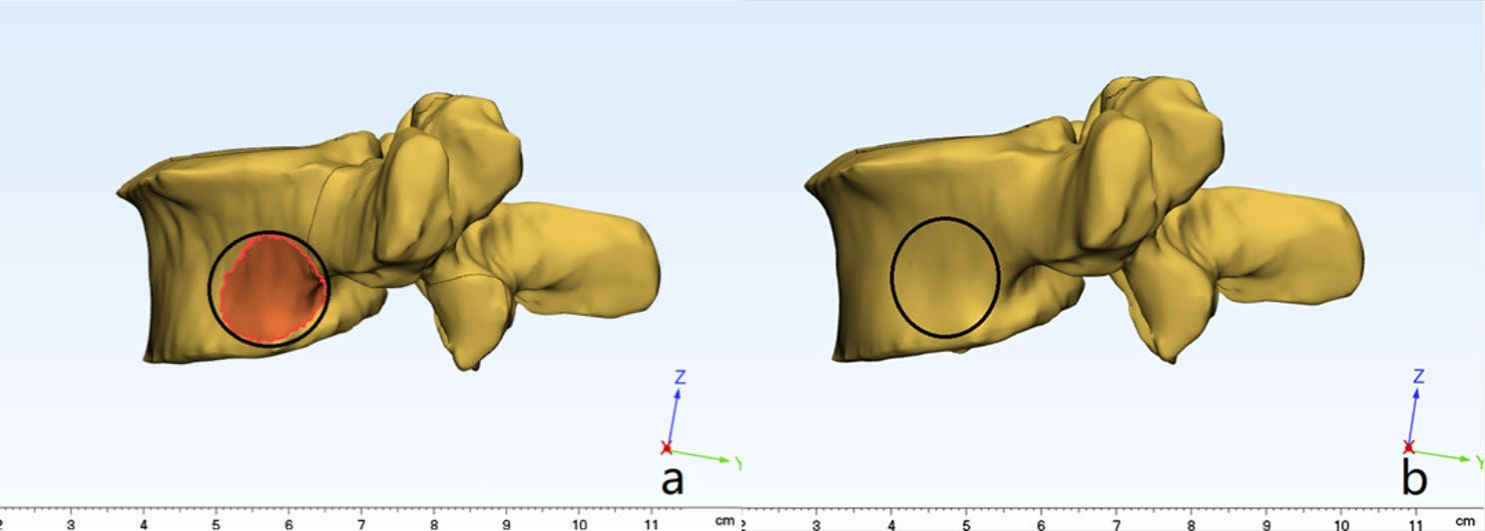
Smoothing of the established L1-S1 3D model. **a**: before smoothing; **b**: after smoothing

Through the smooth and wrap functions in Mimics software, the finite element model was preliminarily processed, and the surface of the model was simplified by increasing the quality of the bone and introducing some irregular structures on the surface. The model was subsequently input into Geomagic Studio software to minimize the noise points of the point cloud, fill the ineffective cavities on the surface of the finite element model, remove the characteristic burrs and dents, smooth and relax the surface (Fig. 3), and finally obtain the geometric model of the L1-S1 segment for this experiment. The surface was smoothed and relaxed (Fig. 3) to obtain the L1-S1 segment geometry model for this experiment.

#### Segmenting the finite element model and assigning material properties to each tissue

A finite element model of the L1-S1 segment was generated, and the structures of cancellous bone, cortical bone and the posterior part of the vertebral body were established. The complete 3D model was imported into Ansys 18.0 computing software for finite element analysis, and the material properties were added to the material library according to the literature [17–19]. Then, the values were assigned to each structure, and the material parameters are shown in Tables 1 and 2.

**Table 1:** Assignment properties of the constituent structures of the L1-S1 sections

anatomical structure	modulus of elasticity/MPa	Poisson’s ratio
cortical bone	12,000	0.3
cancellous bone	100	0.2
cartilaginous endplate	3500	0.25
normal annulus fibrosus	2.6	0.4
degenerated annulus fibrosus	12.3	0.35
normal nucleus pulposus	1.0	0.49
degenerating nucleus pulposus	1.7	0.4

**Table 2:** Properties of the major ligaments of the L1-S1 segments

ligaments	modulus of elasticity (MPa)		cross-section (mm^2^)	average length (mm)
anterior longitudinal ligament (ALL)	7.8	22.4		20
posterior longitudinal ligament (PLL)	10	7.0		12
ligamentum flavum (LF)	17	14.1		15
intertransverse ligament (ITL)	10	0.6		32
capsular ligament (CL)	7.5	10.5		5
interspinous ligament (ISL)	10	14.1		13
supraspinous ligament (SSL)	8			22

### Simulation of foraminoplasty on the three-dimensional finite element model

Basis on the normal L1-S1 3D finite element model, the following 3D finite element models were established based on the intraoperative situation by simulating the surgical procedure using Mimics software.

- L4-5disc herniation without surgical treatment model (M1): Approximately 1/4 of the left posterior part of the L4-5 disc was defined as the material property of the degenerated disc, and other tissues such as the L3-4 and L5-S1 discs were defined as normal properties.
- 5 mm foraminoplasty model (M2): The fibrous annulus and nucleus pulposus of the left posterior 1/4 of the L4-5 intervertebral disc were removed to simulate resection of the protruding disc and replaced with scar tissue. The tip of the left superior articular process of L5 was used as the puncture point, 5 mm of bone was removed from the tip of the foraminoplasty site at an angle of approximately 30 degrees from the coronal plane to simulate the removal of part of the bone of the superior articular process, the foramen was enlarged, and foraminoplasty was performed. This model simulated the intraoperative removal of the L5 superior articular process using a conventional 5 mm-diameter trephine under ordinary endoscopy.
- 8.5 mm foraminoplasty model (M3): In the same way as above, this model simulated the intraoperative removal of the L5 superior articular process using a 8.5 mm-diameter trephine under large-channel endoscopy.

### Loading conditions and observables

When the model is in the neutral position, the bottom of S1 is fixedly constrained, and the upper surface of the L1 endplate is not constrained as the location of pressure and moment application. When setting the magnitude and direction of the load, the ‘Component’ method is selected to set the load, which simulates the stress on the lumbar vertebrae caused by the weight of the human body under different conditions, so that the load is uniformly transmitted to the nodes on the surface. Pressure and moment were applied to the upper surface of the L1 vertebral body. Pressure and moment were 1175 N and 7.5 N⋅m in flexion, 500 N and 7.5 N⋅m in posterior extension, 700 N and 7.8 N⋅m in lateral flexion, and 720 N and 5.5 N⋅m in rotation [20–22], simulating the lumbar spine under six working conditions, including forward flexion, backward extension, left and right lateral bending and left and right rotation.

Definition of contact surfaces and interactions: After the model was assembled, the contact surfaces were defined according to the anatomical situation. For the relative sliding between the lumbar disc surfaces and the upper and lower endplates of each vertebral body, contact was defined as a “tie” pattern, with the upper and lower endplates of each vertebral body serving as the master surfaces, and the upper and lower surfaces of the disc serving as the follower surfaces. The FJ was set as a micromanipulation joint, and contact was defined as limited sliding contact with a friction coefficient of 0.1, with the next vertebral body’s upper articular process serving as the master surface.

Changes in the range of motion of the L4-5 segment under forward flexion, backward extension, left and right lateral bending and left and right rotation were observed in the three models. The stress changes in L3-4, L4-5 and L5-S1 discs of the three models were observed. The stress changes in L4-5 FJs were observed in the three models. For the recording of the simulation results, according to previous methods reported in the literature, data were collected in the region of maximum stress or displacement, and five points in the collection region were randomly selected and represented by the mean.

### Statistical analyses

Statistical processing was carried out using SPSS 23.0 software, and measurement data are presented as the means ± standard deviations (‾*x* ± s). Comparisons of data between multiple groups were performed using one-way analysis of variance (ANOVA), and if the results of the analyses were overall different, then two-by-two comparisons were performed using the least significant difference (LSD) method. *P* < 0.05 was considered to indicate statistical significance.

## Results

### Normal 3D finite element modeling and validation

**Fig. 4:**
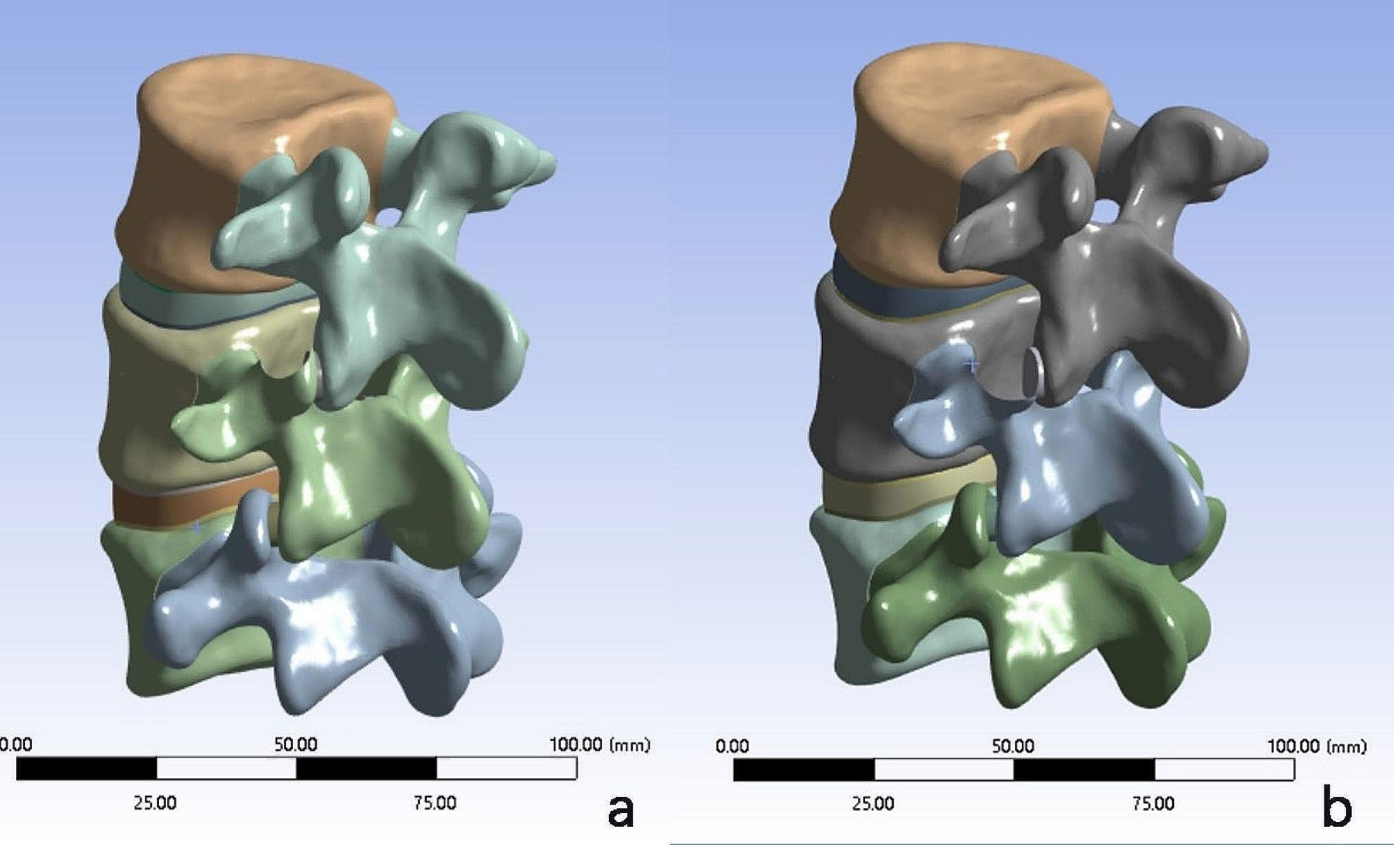
Simulation of foraminoplasty on L5 superior articular process under ordinary and large-channel endoscopy

We established the L4-5disc herniation model (M1), 5 mm foraminoplasty model (M2) and 8.5 mm foraminoplasty model (M3) (Fig. 4), and compared with the model of Shim [23] (Table 3), we found that the range of motion (ROM) of the joints was in a reasonable range, which proved the validity of the present finite element model.

**Table 3:** Range of motion of the normal L1-S1 finite element model L3-4 and L4-5 segments under different working conditions ($\bar{x} \pm \mathrm{s}$)

Working condition	Sections L3-4		Section L4-5	
	This study	Shim’s research	This study	Shim’s research
Forward flexion	3.346	4.2 ± 0.8	5.873	5.4 ± 0.9
Backward extension	2.636	2.9 ± 0.5	3.203	2.9 ± 0.5
Left rotation	2.727	2.8 ± 0.6	3.890	3.8 ± 1.0
Right rotation	2.900	2.8 ± 0.6	4.023	3.8 ± 1.0
Left bending	4.154	3.5 ± 1.0	3.842	4.4 ± 1.1
Right bending	4.301	3.5 ± 1.0	3.625	4.4 ± 1.1

### Mechanical changes after stress loading in the three models

#### Displacement analysis of L4 vertebra

**Fig. 5:**
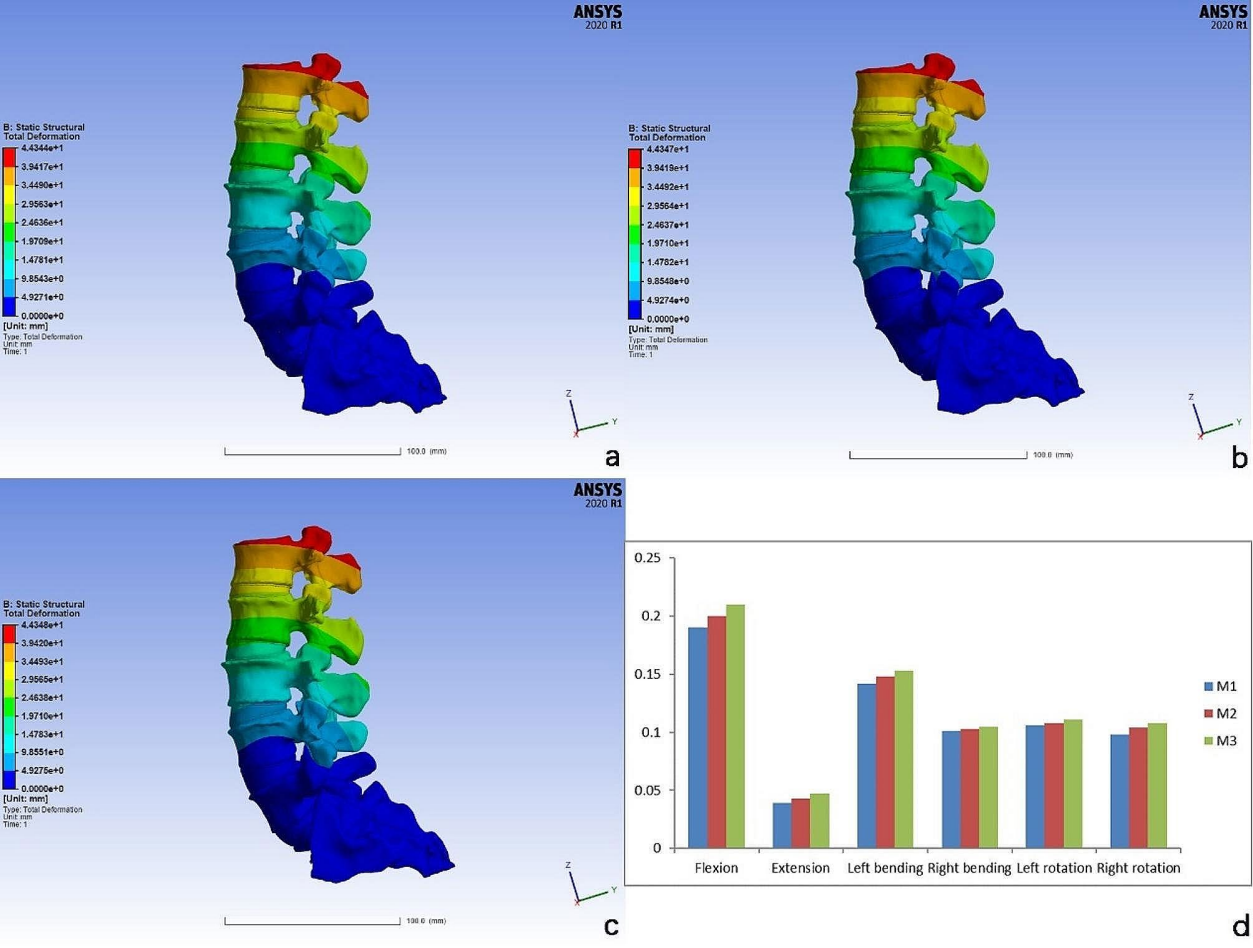
Cloud plots of relative displacements of L4 vertebra under vertical stress (**a-c**) and histograms of the degree of relative displacement of L4 vertebra (**d**)

As shown in Fig. 5, the differences in the degree of relative displacement of the L4 vertebra among the three models under the six working conditions were not statistically significant (*P* > 0.05).

#### Stress analysis of L4-5 facet joints

**Fig. 6:**
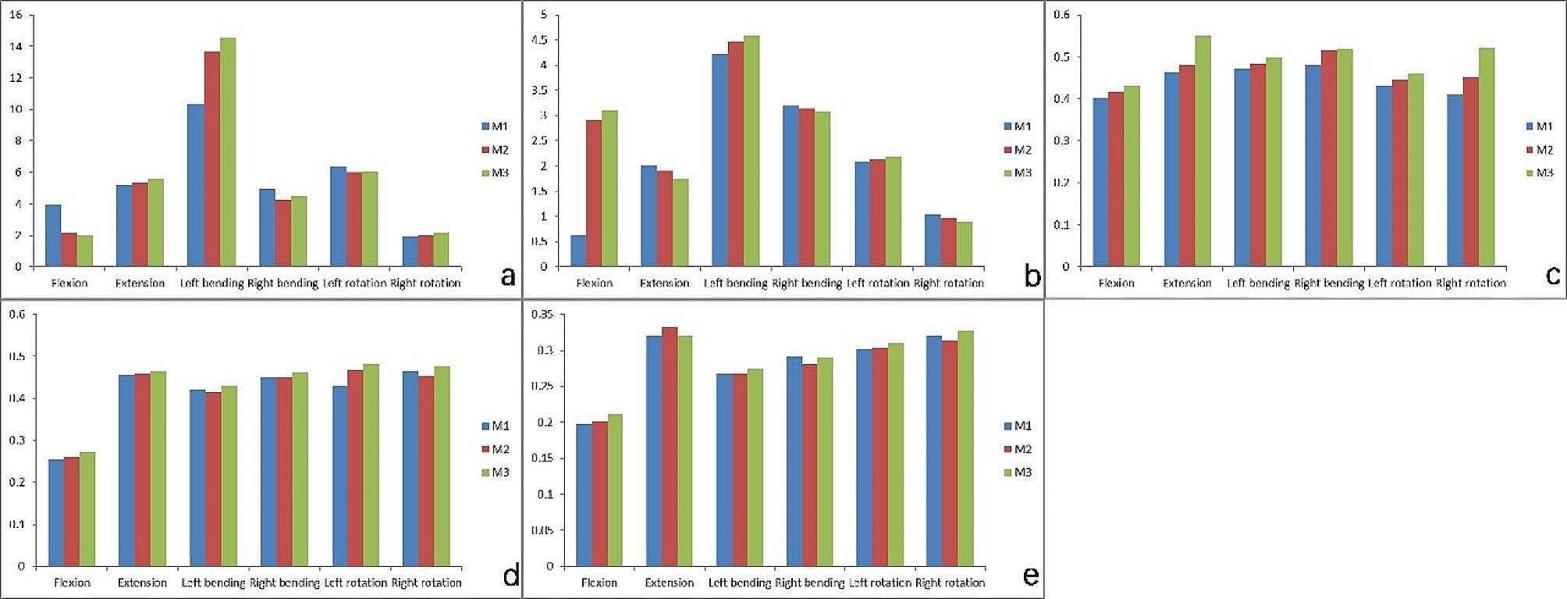
Histograms of the stress distribution in facet joints and intervertebral discs under six working conditions. **a**: stress distribution in the left FJ of L4-5; **b**: stress distribution in the right FJ of L4-5; **c**: stress distribution in L4-5 disc; **d**: stress distribution in L3-4 disc; **e**: stress distribution in L5-S1 disc

Compared with those in the M1 model, the stresses in the L4-5 left FJ decreased under forward flexion conditions in the M2 and M3 models (*P* < 0.05), but there was no significant difference between the M2 and M3 models (*P* > 0.05). There was no significant difference in the L4-5 left FJ stress between the M2 and M3 models under left lateral bending condition (*P* > 0.05), but both were greater than that of the M1 model (*P* < 0.05). Under posterior extension, right lateral bending, left rotation and right rotation working conditions, there was no statistically significant difference in L4-5 left FJ stress among the three models (*P* > 0.05). In the M2 and M3 models, the stress of the L4-5 left FJ was significantly greater than that in M1 in left lateral bending, left rotation, right lateral bending, and right rotation (Fig. 6a).

Under forward flexion condition, the L4-5 right FJ stress increased significantly in the M2 and M3 models compared with that of the M1 model (*P* < 0.05), but there was no significant difference between the M2 and M3 models (*P* > 0.05). The differences in L4-5 right FJ stress were not statistically significant among the three models under posterior extension, right and left lateral bending and right rotation working conditions (*P* > 0.05). Under left rotation condition, the L4-5 right FJ stress in M3 was greater than that in M2, and the stress in M2 was greater than that in M1, and the differences were statistically significant (*P* < 0.05). In the M2 and M3 models, the L4-5 right FJ stress was significantly greater than that in M1 under right lateral bending, right rotation, left lateral bending, and left rotation conditions (Fig. 6b).

#### Stress analysis of L4-5 discs

The L4-5 disc stress in the M2 and M3 models were greater than that in the M1 model under forward flexion, left rotation, and right and left lateral bending conditions, but the differences were not statistically significant (*P* > 0.05). L4-5 disc stress was greater in the M3 model than those in the M1 and M2 models under posterior extension condition (*P* < 0.05), whereas there was no significant difference between the M1 and M2 models (*P* > 0.05). Under right rotation condition, the L4-5 disc stress in the M3 model was greater than that in the M2 model, and the L4-5 disc stress in the M2 model than that in the M1 model, and the differences were statistically significant (*P* < 0.05) (Fig. 6c).

#### Stress analyses of the adjacent segmental discs

There was no significant difference in the stress distribution of L3-4 and L5-S1 discs among the three models under six working conditions (Fig. 6d and e).

## Discussion

The FEA method can simulate the human body and analyze the biomechanical changes associated with different surgical methods on various parts of the spine and is widely used in the field of spinal surgery [24–27]. The L4-5 segment is the segment with the highest incidence of LDH, so in the present study, the L4-5 segment was used as the target segment, and the FEA method was used to establish a normal 3D finite element model of the L1-S1 segment. Based on this model, an intraoperative foraminoplasty model was established to investigate the effects of intraoperative foraminoplasty on lumbar spine biomechanics. A foraminoplasty model was established based on this model to investigate the effect of intervertebral foraminoscopy on the biomechanics of the lumbar spine in patients after foraminoplasty. Based on this model, a large-channel-based foraminoplasty model was established to investigate the effects of intraoperative foraminoplasty on lumbar biomechanics, providing a theoretical basis for improvement of treatment strategies and prediction of patient prognosis.

The first step after establishing a 3D finite element model is to verify the validity of the model, which is usually performed by comparing the experimental results with similar 3D finite element models from previous literature [28–30]. A comparison of our established finite element model with the results of Shim et al. [23] shows that the ROM of our model is within the error range reported in the literature and is comparable, indicating the validity of the finite element modeling method and material assignment in this paper, which can be used for biomechanical analysis. The modeling methods and loading constraints used in this study are the same as those used in previous studies, with only the individual samples differing, and the results are plausible from the point of view of a qualitative comparative study. The diversity of the individual morphology and material properties of vertebral cancellous bone means that finite element models do not exactly match the results of computer simulations with those of in vivo experiments [31, 32]. Most studies including the present study assign values based on previous studies, rather than patient-specific values, which is a limitation. The reason for this difference is the inconsistency in the sources used to construct the finite element models, and although the ideal approach would be to use the same in vitro experimental subject and finite element model object, this approach is essentially impossible for any experiment due to ethical issues. The advantage of using cadaveric samples for modeling is that the samples can be dissected and directly validated for each tissue, and the disadvantage is that the metrics in the physiological state are not available. Although the reconstruction of the human spine model can be achieved using cadaveric samples, it cannot be validated against homologous cadaveric samples, so the method of validating the finite element model is mainly to compare it with the test data of previous cadaveric specimens.

During TESSYS, depending on the size of the intervertebral foramen and the location of the herniated disc, foraminoplasty on one side is usually required to enlarge the foramen, a procedure that can injure the FJ to varying degrees. Previous studies have reported on the effect of conventional open surgery on FJs. Shah et al. [33] reported that 33–35% of FJs are injured during lumbar nailing via the transosseous interspace approach, whereas Monshirfar et al. [34] analyzed the probability of injury to the FJ during pedicle screw placement via the posterior median approach, and the presence of an injury to the FJ was detected on postoperative radiographs in approximately 15% of the patients. After we established a finite element model of intraoperative foraminoplasty, we analyzed the degree of displacement of the L4 vertebral body under different working conditions, and found that there was no significant difference in L4 vertebral body displacement among the three models, which suggests that intraoperative foraminoplasty is relatively safe and generally does not cause lumbar instability in the postoperative period.

Stress analyses of the L4-5 FJ in the three models revealed that after foraminoplasty, the stresses in the L4-5 bilateral FJ in the left lateral flexion condition increased compared with those in the preoperative condition in all patients and that the 8.5 mm foraminoplasty had a greater effect on the right FJ than did the 5 mm foraminoplasty. In two foraminoplasty models, the stress in the bilateral FJ of L4-5 was significantly greater in left lateral bending or left rotation than in right lateral benging or right rotation, and the magnitude of the increase was greater than that in the unoperated model, which suggests that foraminoplasty with an ipsilateral disc injury will lead to increased stress in the contralateral FJ. The larger the extent of foraminoplasty is, the greater the increased stress in the contralateral FJ. This finding suggests that it is better to minimize the removel of the FJ when performing foraminoplasty. Stress analysis of the L4-5 intervertebral discs of the three models showed that L4-5 intervertebral disc stress was greater in the M3 model than that in the M2 model under conditions of posterior extension and right rotation, which indicated that a large range of foraminoplasty might aggravate the degeneration of the intervertebral discs of the segments compared with a small range of foraminoplasty. In contrast, there was no significant difference in the distribution of L3-4 or L5-S1 disc stresses among the three models under various working conditions, suggesting that foraminoplasty had no significant effect on disc degeneration in adjacent segments. This is an advantage of nonfusion surgery over fusion surgery, as lumbar fusion surgery mostly accelerates the degeneration of neighboring segments [35–38].

There were several limitations in this study. First, the finite element models established in this study are similar to those that have been well-validated in previous studies, but they all simplify the physiological contraction force of the lumbar spine to varying degrees, especially simplifying the muscles connected to the lumbar spine and the weight of the upper body, which is still somewhat different from that of a real human body. Second, t FEA is an instant loading analysis method, which cannot systematically analyze the effect of fatigue loading on lumbar biomechanics. Third, the geometric characteristics of the intervertebral disc have a strong influence on the biomechanical behaviour of the segment [39], but in the current study, only a single geometry has been considered for disc modelling. Furthermore, the model did not take into consideration many patient-specific parameters, such as various level of degeneration and bulging, nutrient supply and biochemical alterations in intervertebral disc as proposed in previous study [40, 41]. In future, individualized computational modeling, which may incorporate patient-specific geometry and/or tissue properties, is needed to improve treatment strategies and patient prognosis.The actual clinical situation is complex and variable, and the repeated accumulation of stress in the lumbar spine after disc removal and foraminoplasty may accelerate the degeneration of the injured area.

The outcomes of the present study indicated that foraminoplasty using large-channel endoscopy increased the stress on the facet joints and disc of the surgical segment, which suggested unnecessary and excessive resection should be avoided in PTED to minimize biomechanical disruption. Further research should integrate clinical outcomes, a broader range of surgical scenarios and patient-specific models to valid the connection of these findings to the clinical outcomes and assess how individual variations in spinal anatomy affect the biomechanical and clinical outcomes, which could enhance the utility of these findings in patient selection, surgical planning, and the improvement of surgical procedures and patient prognosis.

## Data Availability

No datasets were generated or analysed during the current study.

## Abbreviations

LDH: Lumbar Disc Herniation
FJ: Facet Joint
PETD: Percutaneous Endoscopic Transforaminal Discectomy
FSU: Functional Spinal Unit
FEA: Finite Element Analysis
PLIF: Posterior Lumbar Interbody Fusion
